# Type 2 diabetes subgroups and potential medication strategies in relation to effects on insulin resistance and beta-cell function: A step toward personalised diabetes treatment?

**DOI:** 10.1016/j.molmet.2020.101158

**Published:** 2020-12-30

**Authors:** Anna Veelen, Edmundo Erazo-Tapia, Jan Oscarsson, Patrick Schrauwen

**Affiliations:** 1 Department of Nutrition and Movement Sciences, NUTRIM School for Nutrition and Translational Research in Metabolism, Maastricht University Medical Centre, 6200 MD, Maastricht, the Netherlands; 2 Biopharmaceuticals R&D, Late-Stage Development, Cardiovascular, Renal, and Metabolism, AstraZeneca, Gothenburg, Sweden

**Keywords:** Type 2 diabetes, Personalised medicine, Diabetes classification, β-cell function, Insulin sensitivity

## Abstract

**Background:**

Type 2 diabetes is a syndrome defined by hyperglycaemia that is the result of various degrees of pancreatic β-cell failure and reduced insulin sensitivity. Although diabetes can be caused by multiple metabolic dysfunctions, most patients are defined as having either type 1 or type 2 diabetes. Recently, Ahlqvist and colleagues proposed a new method of classifying patients with adult-onset diabetes, considering the heterogenous metabolic phenotype of the disease. This new classification system could be useful for more personalised treatment based on the underlying metabolic disruption of the disease, although to date no prospective intervention studies have generated data to support such a claim.

**Scope of Review:**

In this review, we first provide a short overview of the phenotype and pathogenesis of type 2 diabetes and discuss the current and new classification systems. We then review the effects of different anti-diabetic medication classes on insulin sensitivity and β-cell function and discuss future treatment strategies based on the subgroups proposed by Ahlqvist et al.

**Major Conclusions:**

The proposed novel type 2 diabetes subgroups provide an interesting concept that could lead to a better understanding of the pathophysiology of the broad group of type 2 diabetes, paving the way for personalised treatment choices based on understanding the root cause of the disease. We conclude that the novel subgroups of adult-onset diabetes would benefit from anti-diabetic medications that take into account the main pathophysiology of the disease and thereby prevent end-organ damage. However, we are only beginning to address the personalised treatment of type 2 diabetes, and studies investigating the effects of current and novel drugs in subgroups with different metabolic phenotypes are needed to develop personalised treatment of the syndrome

## Highlights

• Novel subgroups of type 2 diabetes provide a concept that could lead to a better understanding of its pathophysiology.
• Treatment strategies would benefit from anti-diabetic medications that influence the main pathophysiology of diabetes.
• Here, we review different anti-diabetic medications classes affecting insulin sensitivity and β-cell function.
• We suggest that future treatment strategies could benefit by taking into account subgroups provided by Ahlqvist et al.

## Abbreviations

ANS: autonomous nervous system
ASCVD: atherosclerotic cardiovascular disease
CKD: chronic kidney disease
EGP: endogenous glucose production
EMCL: extramyocellular lipid
ER: endoplasmic reticulum
GADA: glutamic acid decarboxylase antibodies
GIP: glucose-dependent insulinotropic polypeptide
HF: heart failure
IMCL: intramyocellular lipid
LADA: latent autoimmune diabetes in adults
MARD: mild age-related diabetes
MOD: mild obesity-related diabetes
MRS: magnetic resonance spectroscopy
NAFLD: non-alcoholic fatty liver disease
NASH: non-alcoholic steatohepatitis
iNKT cell: invariant natural killer T-cell
PPAR: peroxisome proliferator-activated receptor
SAID: severe autoimmune diabetes
SIDD: severe insulin-deficient diabetes
SIRD: severe insulin-resistant diabetes

## 1 Introduction

Type 2 diabetes mellitus (T2D) is a global health problem that according to the International Diabetes Federation will affect 700 million people by 2045 [1,2]. Treatment requires a multidisciplinary approach to prevent and decrease the risk of complications. Glucose-lowering medication is a key element for controlling blood glucose levels. Increased blood glucose levels in T2D are explained by a combination of insulin resistance and reduced β-cell function. In some T2D patients, insulin resistance predominates and in other patients, reduced insulin secretion is the main dysfunction. The mechanisms underlying β-cell failure and reduced insulin sensitivity are multifaceted. Despite these multifactorial aspects of the disease, treatment options remain relatively limited and are often not personalised toward the underlying causes of hyperglycaemia. Importantly, T2D is a systemic syndrome affecting almost all of the tissues in the body, and the disease is associated with an increased risk of many diseases including cardiovascular (CV) diseases, kidney disease, non-alcoholic fatty liver disease (NAFLD), Alzheimer's disease, and various cancers. To date, none of the glucose-lowering medications have had any major impact on end-organ protection. However, recent studies have shown that sodium-glucose cotransporter 2 (SGLT2) inhibitors and glucagon-like peptide-1 receptor agonist (GLP-1RA) reduce the risk of CV disease showing end-organ protection beyond glucose lowering. In this review, we aim to provide a short overview of the pathogenesis and classification of T2D, effects of medication classes on insulin sensitivity and β-cell function and aim to provide future treatment perspectives.

## 2 Pathogenesis of diabetes

Type 2 diabetes is a disease that includes multiple metabolic dysfunctions characterised by hyperglycaemia that is the result of various degrees of pancreatic β-cell failure and reduced insulin sensitivity. Risk factors for developing T2D include obesity, a sedentary lifestyle, and associated insulin resistance. However, most obese and insulin-resistant individuals never develop T2D, which is explained by strong genetic components associated with T2D. As presented by DeFronzo in 1988 [3], the development from impaired glucose tolerance (IGT) to T2D is mainly the result of decreased β-cell function and not due to altered insulin-mediated glucose uptake, whereas insulin resistance is often already present before hyperglycaemia and an increase in HbA1c occurs. However, it should be noted that treatment of insulin resistance would reduce the β-cell burden and improve hyperglycaemia. The risk of developing T2D is strongly inherited, and many genetic associations have been described, although they explain just a fraction of the genetic association [4]. Most of the genetic associations have been ascribed to β-cell function and very few have been linked to insulin resistance [4], although this may be partly because no good measures of insulin sensitivity are available in large cohorts.

In T2D, β-cell failure has been shown to be associated with a 24–65% loss of β-cell mass and a 50–97% loss of insulin secretory capacity of β-cells [5]. Pancreatic β-cells initially overcome insulin resistance in peripheral tissues by producing more insulin, leading to supraphysiological insulin concentrations. Over time, β-cell failure occurs, leading to elevated postprandial and fasting glucose levels despite continued hyperinsulinaemia. Mechanisms that have been associated with β-cell failure include insulin resistance, glucotoxicity, lipotoxicity, β-cell senescence [6,7], dedifferentiation [8], and/or apoptosis [9,10]. First-degree relatives of T2D have dysregulated insulin secretion, with less regular pulsatility of insulin secretion [11]. This change in insulin pulsatility may result in the downregulation of insulin action and indicates an interaction between dysregulated β-cell function and worsening of insulin resistance [12]. Therefore, it is not fully clear if insulin resistance proceeds β-cell failure in all individuals who develop T2D.

**Figure 1:**
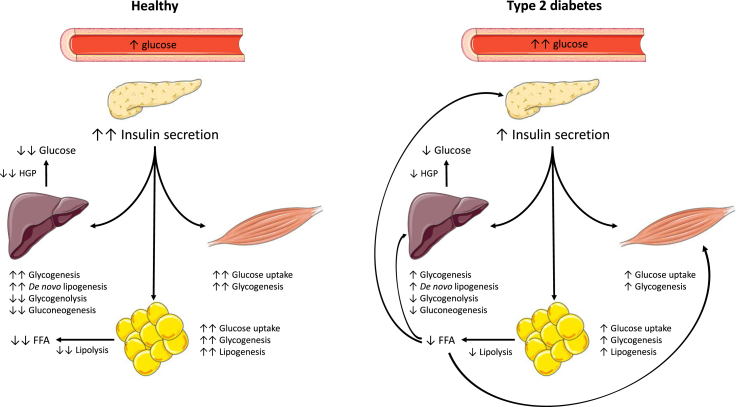
Action of insulin in the postprandial state in healthy and type 2 diabetes conditions. Increasing blood glucose will lead to the secretion of insulin. Insulin stimulates glucose uptake in skeletal muscle and white adipose tissue and suppresses lipolysis in white adipose tissue, leading to a reduction in circulatory free fatty acid (FFA) levels. In the liver, insulin and reduced adipose lipolysis suppresses hepatic glucose production (HGP) via a combination of reductions in gluconeogenesis and glycogenolysis and stimulation of glycogen storage. The combined action of glucose uptake and reduction in HGP contributes to plasma glucose control. In type 2 diabetes, glucose-induced insulin secretion is not sufficient due to reduced β-cell function and insulin-stimulated glucose uptake in muscle and white adipose tissue (WAT) as well as insulin-stimulated suppression of HGP is blunted. Insulin resistance in WAT also leads to blunted suppression of lipolysis by insulin, producing higher FFA levels that subsequently negatively affect skeletal muscle and HGP. FFA, free fatty acids; HGP, hepatic glucose production.

The other major hallmark in the development of T2D is the gradual development of whole-body and peripheral insulin resistance. As skeletal muscle, the largest organ in the body, is responsible for approximately ∼85% of postprandial glucose uptake, skeletal muscle insulin resistance contributes to the development of hyperglycaemia [13]. In skeletal muscle, insulin resistance is characterised by reduced intracellular insulin-stimulated glucose uptake and handling due to reduced insulin-induced GLUT4 translocation to the cell membrane and subsequent glycogen synthesis (Figure 1) [14].

In addition to skeletal muscle, liver insulin resistance results in elevated basal endogenous glucose production (EGP) and reduced insulin suppression of EGP, further contributing to higher plasma glucose concentrations (Figure 1) [9]. Adipose tissue insulin resistance contributes to hyperglycaemia by reduced glucose uptake, although adipose tissue glucose uptake is generally considered relatively low in humans [15]. However, adipose tissue insulin resistance also leads to reduced inhibition of lipolysis by insulin, which results in elevated free fatty acid (FFA) levels in the blood (Figure 1) [16,17]. High circulatory FFA can contribute to skeletal muscle insulin resistance. Furthermore, higher rates of lipolysis also cause higher levels of glycerol, which are considered an important source of gluconeogenesis and EGP [18]. Please see Figure 1 for an illustration of common changes in postprandial insulin action in type 2 diabetes.

## 3 Underlying causes of the development of β-cell failure, insulin resistance, and T2D

T2D is strongly associated with obesity, and ∼90% of all T2D patients are overweight or obese. Expansion of fat mass serves to ensure the storage of excess nutrients/energy; however, when adipose tissues expandability becomes limiting or dysfunctional [19], circulating FFA and elevated uptake of FFA in the liver and skeletal muscle can occur, where they can compete with glucose for substrate oxidation and according to the Randle cycle can contribute to insulin resistance [20]. FFA can also accumulate in non-adipose tissues, and ectopic fat accumulation has been shown to be a crucial factor in the development of insulin resistance in the liver and skeletal muscle, mainly due to the interference of diacylglycerol and ceramides (among others) with the insulin-signalling pathway [[21], [22], [23]]. Increased FFA uptake is also associated with oxidative stress, inflammation, and cell death. Lipotoxicity can occur in a range of tissues such as skeletal muscle, heart, arteries, pancreas, and liver, creating different phenotypes/end-organ damage among patients depending on which organs are most affected. In muscle, fat accumulation interferes with insulin-stimulated GLUT4 translocation, and in the liver, non-alcoholic fatty liver (NAFL) is associated with hepatic insulin resistance and enhanced production of VLDL-TG that contributes to the development of atherogenic/diabetic dyslipidaemia [24,25]. As previously mentioned, the development of hepatic insulin resistance could also be due to deficient pulsatile insulin delivery into the hepatic portal vein and eventually to the hepatocytes [12]. This hypothesis suggests that dysregulated insulin delivery, which is present in T2D, could lead to dysregulation of hepatic lipid metabolism or selective insulin resistance through FoxO1, contributing to the accumulation of lipids [26]. Selective insulin resistance refers to the pathological state in which insulin does not decrease hepatic glucose production, but insulin stimulation of *de novo* lipogenesis via activation of SREBP-1c is unaffected and further increased due to the associated hyperinsulinaemia, leading to hepatic fat accumulation [27]. In the pancreas, β-cell exposure to chronic high levels of FFA leads to endoplasmic reticulum (ER) stress and mitochondrial dysfunction, which can result in cell damage and eventually impaired insulin secretion [28].

Chronic hyperglycaemia has also been shown to exert toxic effects on β-cells and other tissues, a phenomenon termed glucotoxicity. Glucotoxicity contributes to β-cell failure and reduced insulin sensitivity in the liver via different processes, such as ER stress, mitochondrial dysfunction, oxidative stress, and inflammation [10,29]. In addition to chronic hyperglycaemia, glycogen storage in β-cells has been shown to be associated with apoptosis [30]. Whether glucotoxicity also has effects on skeletal muscle insulin sensitivity is still under debate and not fully understood [14].

Apart from obesity, age is another determinant of the development of T2D, which has also long been considered a disease associated with accelerated ageing. Wijsman et al. [31] showed that familial longevity was characterised by better insulin sensitivity compared to a group with same age, sex, and body composition. With age, a decrease in physical activity and muscle mass is often observed, factors that directly contribute to the development of skeletal muscle insulin resistance. In addition, ageing is often associated with an increase in fat mass that can contribute to the development of lipotoxicity and insulin resistance. Cellular stress responses can lead to a state of cellular senescence characterised by cell-cycle arrest, resistance to apoptosis, and a senescence-associated secretory phenotype (SASP), which negatively influence organ functions. It was shown that insulin resistance accelerated β-cell senescence in human islets (Aguayo-Mazzucato). Moreover, in a mouse model of type 1 diabetes, it was shown that elimination of senescent cells halted immune-mediated β-cell destruction and prevented diabetes [32]. Thus, both improved insulin sensitivity and enhanced apoptosis of senescent islet cells could improve β-cell function.

The Baltimore Longitudinal Study of Aging showed that insulin secretion decreases with age independent of BMI and adipose tissue distribution [33]. The latter could explain why the prevalence of T2D is associated with increasing age in the population.

As previously stated, most insulin-resistant people do not develop T2D, and genetic components could explain why some insulin-resistant individuals develop T2D. Genome-wide association analyses have identified single-nucleotide polymorphisms (SNPs) that are associated with the function of the β-cells. Some of these genetic variants are located over 40 loci and can increase the risk of T2D. Although more than 400 gene variants have been associated with the presence of T2D, the currently identified variants account for only 10% of the genetic influence for the risk of developing T2D [34]. In contrast, maturity-onset diabetes in the young is monogenic diabetes and accounts for 2–5% of all diabetes patients [35].

## 4 Classification of diabetes

In 1979, an international work group established a new classification system that included type 1 diabetes mellitus (T1D), T2D, and gestational diabetes [36]. They also added an IGT group: people who did not meet the criteria for diabetes mellitus but have elevated fasting and 2-hour glucose values. In 1997, the classification system was reviewed again and the IGT group was split in two: impaired fasting glucose (IFG) and IGT [37].

More than 40 years after the classification system was first suggested, knowledge about the complexity of diabetes pathophysiology has increased. However, there are still only two major classifications: T1D and T2D. At present, with the call for more a personalised medication strategy, a more refined classification system would be helpful to develop novel drugs correcting the root cause of the syndrome as well as prescribing the best current medication to prevent disease progression and end-organ damage.

In 2018, Ahlqvist et al. [38] suggested a new classification system of adult-onset diabetes, which, at least partly, considers the heterogeneous phenotype of T2D. In their subgroup classification, adult-onset diabetes is classified into five subgroups or clusters using 6 fairly common measures that can be obtained in clinical care: BMI, age at diagnosis, HbA1c, glutamic acid decarboxylase antibodies (GADA), and homeostasis model assessment 2 (HOMA2) to estimate β-cell function (HOMA2-B) and insulin resistance (HOMA2-IR) based on fasting glucose and C-peptide concentrations. Data-driven non-supervised cluster analysis was conducted using large Swedish and Finnish cohorts that included all new incidents of adult-onset diabetes.

**Figure 2:**
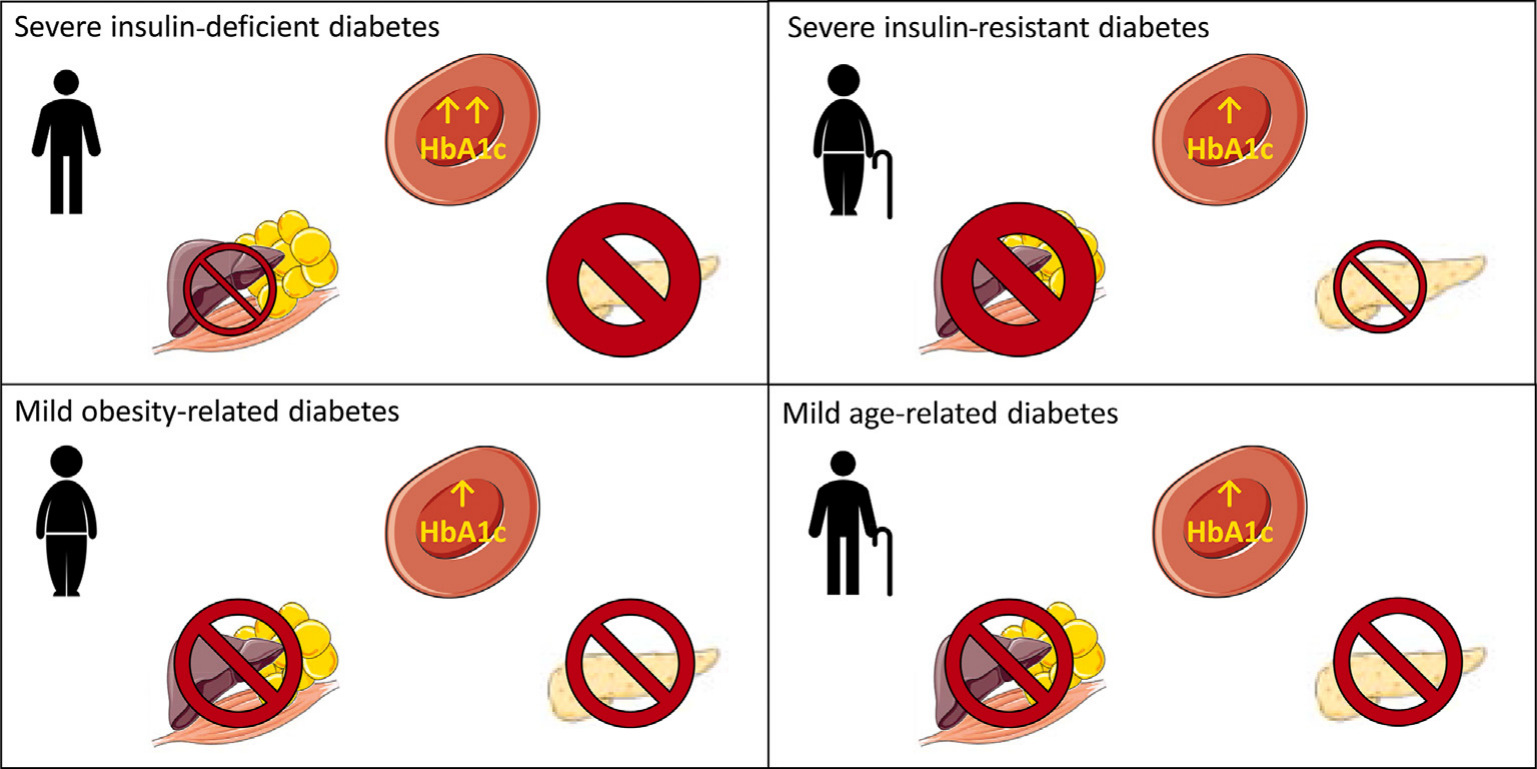
Visual representation of the characteristics of the subgroups as suggested by Ahlqvist et al. [38]. Severe insulin-deficient diabetes (SIDD) is characterised by a relatively low age and BMI, a high HbA1c, less marked insulin resistance, but severe β-cell insulin deficiency. Severe insulin-resistant diabetes (SIRD) is characterised by a relatively high age and BMI, a relatively low HbA1c, severe insulin resistance, but no insulin deficiency. Mild obesity-related diabetes (MOD) is characterised by a relatively low age at diagnosis, a high BMI, relatively low HbA1c, and mild insulin resistance and insulin deficiency. Mild age-related diabetes (MARD) is characterised by a high age at diagnosis, a relatively low BMI, and mild insulin resistance and insulin deficiency. More severe insulin resistance/deficiency is indicated with a larger stop sign.

This data-driven cluster analysis concluded 5 novel subgroups for newly diagnosed adult-onset diabetes based on the aforementioned variables: severe autoimmune diabetes (SAID), severe insulin-deficient diabetes (SIDD), severe insulin-resistant diabetes (SIRD), mild obesity-related diabetes (MOD), and mild age-related diabetes (MARD) (Figure 2).

SAID and SIDD were both characterised by earlier-onset diabetes, a relatively low BMI, poor metabolic control (high HbA1c), and insulin deficiency (determined by a low HOMA2-B index). The difference between SAID and SIDD is the presence of glutamic acid decarboxylase antibodies in SAID but not in SIDD. Severe autoimmune diabetes (SAID) overlaps with both T1D and latent autoimmune diabetes in adults (LADA). The latter share genetic features with T1D, but in a clinical setting, they often share characteristics of T2D patients and therefore are often diagnosed as T2D. Applying the same clustering system in an independent German cohort revealed that patients allocated to the SIDD group also showed signs of autoimmunity [39].

SIRD is characterised by a higher BMI (overweight to obese) and marked insulin resistance (determined by a high HOMA2-IR index). In SIRD, β-cell function is less impaired than in SAID and SIDD (high HOMA2-B index) and HbA1c levels are lower. Both SIDD and SIRD were previously diagnosed as T2D and represent very different forms of severe T2D.

Mild obesity-related diabetes (MOD) and mild age-related diabetes (MARD) are characterised by relatively mild insulin resistance (HOMA2-IR lower than SIRD) and mild insulin deficiency (HOMA2-B higher than SAID and SIDD, but lower than SIRD). The difference between MOD and MARD is based on the age at diagnosis and BMI; MOD is characterised by a high BMI (obesity), while MARD has a higher age at diagnosis. Thus, SAID constitutes patients that are currently diagnosed with T1D or LADA, while patients in to the other four clusters are currently diagnosed with T2D.

The disease progression and risk of end-organ damage seem to differ by subgroups. SAID and SIDD have a higher HbA1c at baseline and during follow-up compared to the rest of the subgroups also associated with an increased risk of ketoacidosis [38,39]. SIRD is associated with a high prevalence of NAFLD and fibrosis at diagnosis [38,39] as well as diabetic kidney disease and end-stage renal disease [38], but when corrected for baseline kidney function, there was no difference between the different subgroups [40]. In other words, patients with SIRD develop end-organ damage before they are diagnosed with diabetes. In contrast, neuropathy and retinopathy are more often associated with the SIDD group [38,39]. The subgroups also differ by the initial treatment prescribed in the cohort at the time of diagnosis. In the SAID group, 42–67% were on insulin treatment and 29–44% of SIDD patients were on insulin treatment [38,39].

## 5 Current treatment strategies

Although T2D is a heterogenous syndrome as indicated by large inter-individual differences with respect to insulin resistance, β-cell function, and autoimmunity, the current treatment strategies mainly focus on lowering glucose and HbA1c to prevent end-organ damage. As atherosclerotic cardiovascular disease (ASCVD) is still the leading cause of morbidity and mortality among patients with T2D, guidelines clearly indicate to what extent the different medications have proven to reduce the risk of CV events. Other end-organ diseases associated with T2D such as chronic kidney disease (CKD), NAFLD, neuropathy, and retinopathy are also relevant complications to consider when choosing the appropriate therapy for patients with T2D. However, at present, predicting disease progression or risks of end-organ damage in individuals with T2D is not fully understood. It would be better and more cost-effective for patients if there were accurate methods of predicting risks to more aggressively treat patients with a higher risk than those with a lower risk.

Following initial recommendations regarding lifestyle modifications, weight loss, and increased physical activity, patient conditions are the reference elements when choosing an anti-hyperglycaemic medication. Guidelines usually consider elements such as the patient's risk of having a cardiovascular event, weight, and risk of hypoglycaemia when selecting anti-diabetic drugs.

Other elements that drive the decision are the costs of medication and proven efficacy. Therefore, in the latest combined guidelines of the American Diabetes Association (ADA) and European Association for the Study of Diabetes (EASD) of 2020, metformin continues to be the first-line therapy given its unique profile when assessing cost-effectiveness and tolerability [[41], [42], [43]]. The mechanism of action of metformin on glucose control is not fully elucidated and both the liver and intestine have been suggested as the main target tissues; the working mechanism of metformin has been extensively reviewed previously (for further reading, see [44]). Nevertheless, a large proportion of patients will be unable to achieve treatment targets by taking metformin only and will eventually require the addition of a second-line therapy.

The choice of a second-line therapy will depend mainly on the patient having established ASCVD, CKD, or heart failure (HF). If these conditions are not established, the decision is instead based on the risk of adverse events such as hypoglycaemia, weight gain, cost, and patient preferences. However, there is little evidence to guide the choice of a second or even third agent to control glucose homeostasis.

The novel diabetes classification system suggested by Ahlqvist et al. [38] shows the heterogeneity of diabetes and focuses on several different factors, including insulin resistance and β-cell dysfunction. This new classification can help develop a new, more personalised treatment approach by exploring the relationship between anti-hyperglycaemic medications and their effects on the mechanistic causes of T2D. With this classification, it may also follow that patients at a higher risk obtain more aggressive treatment at diagnosis to prevent end-organ damage associated with this subtype of diabetes. At present, there are 5 different classes of second-line anti-hyperglycaemic medications recommended by the ADA and EASD: dipeptidyl peptidase 4 (DPP-4) inhibitors, GLP-1RA, SGLT2 inhibitors, sulfonylureas, and thiazolidinediones. These medications have been commercially successful due to their ability to improve glucose homeostasis and reduce HbA1c but have unique and sometimes incompletely discovered mechanisms of action and improve glucose homeostasis in different ways. This provides opportunities for a more personalised medication treatment strategy. Therefore, in this article, we provide an overview of the proposed working mechanisms of currently prescribed second-line anti-hyperglycaemic medications and review the available clinical data on the effects of these medications on β-cell function and insulin sensitivity.

We suggest potential treatment strategies for the novel SIDD, SIRD, MOD, and MARD subgroups presently comprising the large group of T2D, as these groups may benefit from different treatment medications. Importantly, there is very little data to make the right choices for patients depending on their metabolic phenotype. Therefore, our suggestions are hypothesise generating and should not be regarded as recommendations.

The treatment strategies for the novel SAID subgroup will not be discussed as this group includes T1D and LADA and entails a heterogeneous group that currently requires insulin therapy. Sulfonylureas will not be discussed further in this review because their effects on β-cell function and insulin sensitivity are well established. Treatment with sulfonylureas has no effect on insulin sensitivity but will initially improve β-cell function. After 1–2 years of therapy, HbA1c levels increase again, indicating a worsening of β-cell function [45]. However, it should be noted that sulfonylurea has been shown to have the most beneficial effects on HbA1c in patients with MARD [40], demonstrating that sulfonylureas have a place in the long-term treatment of this T2D subgroup. Insulin therapy will not be discussed as we consider it extensively reviewed and guidelines state when and under what considerations insulin has an advantage over other second-line agents [41]. Medications that improve glucose levels, including insulin therapy, allow the rest of β-cells by compensating for insulin demand to correct hyperglycaemia. The β-cell rest concept is beyond the context of this review, but it is relevant to mention that there is currently no clinical evidence that any medication changes the disease progression in terms of improving β-cell function beyond the acute effects [46,47]. However, it is worth mentioning that insulin use in T2D patients has some disadvantages such as the risk of an increase in weight, which may cause insulin resistance, and insulin therapy in patients with T2D may increase the risk of cardiovascular complications [48].

In the following sections, we focus on potential second-line therapies for T2D and mainly include human trials using hyperinsulinaemic clamps or mixed meal tests whenever possible, as these techniques are regarded as the gold standard methods to assess β-cell function and insulin sensitivity.

### 5.1 Sodium-glucose cotransporter 2 (SGLT2) inhibitors

SGLT2 inhibitors are a novel type of glucose-lowering medications acting on SGLT2, which are expressed in the first segment of the proximal tubule in the kidneys. SGLT2 is responsible for approximately 90% of the glucose reabsorption of the kidneys. Inhibition of SGLT2 results in urinary excretion of 60–80 g of glucose per day, the exact amount depending on plasma glucose concentrations and the glomerular filtration rate, leading to a reduction in HbA1c of 0.6–0.9% and fasting glucose of 1.1–1.9 mmol/L compared to placebo [49].

The mechanism via which SGLT2 inhibitors lower glucose levels is simple and direct, by increased loss of glucose via the urine, a mechanism that is independent of insulin action [[49], [50], [51]]. Glucose and energy loss will trigger adaptive responses that may contribute to the beneficial effect of this group of drugs. SGLT2 inhibitors are associated with reduced body weight [49], lower blood pressure, and positive outcomes on CV death, HF, and progression of CKD [[52], [53], [54]].

#### 5.1.1 SGLT2 inhibitors and β-cell function

Urinary glucose loss may improve β-cell function via reduced glucotoxicity and/or a reduction in excessive insulin secretion due to lower glucose concentrations [55]. Although SGLT2 inhibitors do not directly target β-cells, the effects of SGLT2 inhibition on β-cell function have been investigated in several human intervention studies. Al Jobori et al. and Merovci et al. reported a 2-fold increase in β-cell function measured as improvement of the insulin secretion/insulin resistance index (also called the deposition index: the change in the C-peptide concentration divided by the change in the glucose concentration [ΔC-peptide/Δglucose] divided by the insulin resistance) after 2 weeks of SGLT2 inhibitor treatment in patients with T2D [[56], [57], [58]]. In line, Forst et al. reported two independent studies of improved β-cell function assessed as improvements in the area under the curve for insulin, C-peptide, and the C-peptide/pro-insulin ratio during an hyperglycaemic clamp after 30 days of treatment with SGLT2 inhibitors in patients with T2D co-treated with metformin [59,60].

Several studies showed that treatment with SGLT2 inhibitors improves β-cell glucose sensitivity. Ferrannini et al. [61] reported a 25% increase in β-cell glucose sensitivity after only 48 h of SGLT2 treatment in patients with T2D who were treatment naive or treated with metformin. After 14 days of treatment, improvements in β-cell glucose sensitivity were sustained. Three other studies in patients who were treatment naive or given diet advice, metformin, sulfonylureas, or a combination of metformin and sulfonylureas reported that β-cell glucose sensitivity increased after both 48 h and 14 days of SGLT2 treatment [56,58,62].

As previously described, the progression of diabetes is mainly because of the decrease in β-cell function. This means that the long-term effects of improvements in β-cell function can be monitored as no progression in the deterioration of HbA1c levels. The effect of SGLT2 inhibitors on HbA1c was established in a meta-analysis including 38 studies with a duration of ≥24 weeks conducted by Zaccardi et al. [49]. On average, they reported a HbA1c reduction of 0.6–0.9%. When focused on studies with a long-term duration (≥104 weeks) measuring HbA1c, SGLT2 inhibition produced a sustained reduction of 0.30–1.22% [[63], [64], [65], [66], [67]].

#### 5.1.2 SGLT2 inhibitors and insulin sensitivity

SGLT2 inhibition can lead to improved insulin sensitivity via a reduction in plasma glucose and reduced body weight. Weight loss is generally associated with an improvement in insulin sensitivity and a weight loss of 1.5–2 kg has been reported in patients on SGTL2 inhibitor therapy [49,68]. As discussed to follow, a loss of glucose via the urine may lead to a compensatory stimulation of lipid oxidation in humans, which could impact the distribution of excessive fat mass and reduce ectopic fat stores, which are strongly related to the development of insulin resistance [55].

Several studies investigated the effect of SGLT2 inhibition on peripheral insulin sensitivity [61,69,70]. Ferrannini et al. [61] reported a decrease in total glucose disposal corrected for urinary glucose excretion after acute SGLT2 inhibitor administration, which was sustained after 14 days of treatment in patients with T2D who were either treatment naive or co-treated with metformin. However, despite the reduced glucose disposal predominately caused by a decrease in non-oxidative glucose disposal, peripheral insulin sensitivity estimated by the ratio of the glucose metabolic clearance rate to the mean plasma concentration during a mixed meal test significantly increased after acute administration but the increase did not reach statistical significance after 14 days of treatment. Merovci et al. [69] found similar results using hyperinsulinaemic euglycaemic clamps to assess insulin sensitivity. Fourteen days of SGLT2 inhibitor administration increased insulin-stimulated whole-body glucose disposal corrected for urinary glucose loss from 4.3 ± 0.4 to 5.0 ± 0.4 mg/kg/min, which was a significant increase compared to baseline and placebo (4.0 ± 0.5 to 4.3 ± 0.6 mg/kg/min) in patients with T2D treated with either metformin or a combination of metformin and sulfonylureas. Similarly, after 12 weeks of SGLT2 inhibitor treatment, peripheral insulin sensitivity measured during hyperinsulinaemic euglycaemic clamps improved compared to placebo in patients with T2D co-treated with metformin or a combination of metformin and an insulin secretagogue [70].

Similar results were found in other studies. Thus, in patients with T2D co-treated with either metformin, sulfonylureas, DPP4 inhibitors, or a combination of metformin and sulfonylureas, peripheral insulin sensitivity improved by approximately 16–36% compared to baseline and placebo after SGLT2 inhibitor administration [57,58,71,72]. In contrast, Latva-Rasku et al. [73] did not find an improvement after 8 weeks of SGLT2 inhibition on insulin sensitivity (measured as whole-body insulin-stimulated M values) or skeletal muscle glucose uptake in patients with T2D co-treated with metformin or metformin in combination with DPP-4 inhibitors. The authors indicated that severe insulin resistance among the participants could explain why a relatively lower insulin infusion rate (40 mU/m^2^/min) did not detect a change in M values. Although liver fat content decreased significantly (proton density fat fraction: 3.7%), this reduction in hepatic fat did not lead to an improvement of hepatic insulin sensitivity (measured as suppression of EGP) or enhanced glucose uptake in the liver.

Conversely, several studies reported an increase in EGP after SGLT2 inhibitor administration [61,[69], [70], [71],^74^]. The hepatic and possibly renal production of glucose compensates for approximately one-half of the glucose lost in urine in T2D patients, thereby blunting the decrease in the plasma glucose concentrations [69]. The exact mechanism leading to a compensatory increase in EGP remains unclear. It has been suggested that a decreased insulin:glucagon ratio or autonomic nervous system (ANS)-mediated mechanisms could be involved. Alatrach et al. [75] demonstrated that insulin and glucagon concentrations under glucose clamp conditions (prevention of a decrease in glucose levels) did not differ between subjects receiving SGLT2 inhibitors or placebo, but SGLT2 inhibition caused an increase in EGP in contrast to placebo. This argues against the important role of the insulin:glucagon ratio in mediating the increase in EGP after SGLT2 inhibition. Solis-Herrera et al. and Daniele et al. [76,77] hypothesised that renal ANS afferents are important for increased EGP following SGLT2 inhibition. They investigated the effect of SGLT2 inhibition on EGP in kidney transplant patients with either both residual native kidneys in place or a bilateral nephrectomy. An increase in EGP after SGLT2 inhibitor administration occurred in both patient groups. While the increase in EGP in patients with their native kidneys was comparable to other studies, the increase in EGP was blunted in patients with a bilateral nephrectomy. This finding indicates a role of the kidneys and/or ANS in the increase in EGP; however, the mechanism leading to the increase in EGP following SGLT2 inhibition remains unclear.

SGLT2 inhibition has been reported to result in changed substrate oxidation, which may have favourable effects on insulin sensitivity. Thus, a decrease in glucose oxidation and an increase in lipid oxidation and ketone production have been reported [71,78], which could contribute to improvements in β-cell function and insulin sensitivity by reducing ectopic fat and ameliorating lipotoxicity. However, increased fatty acid oxidation is associated with increased adipose tissue lipolysis and increased flux of fatty acids that would reduce glucose uptake in skeletal muscle, decreasing skeletal muscle insulin-mediated glucose uptake. However, there is limited knowledge about changes in potentially harmful intracellular lipids, and to the best of our knowledge, ectopic fat has been shown to be reduced in the liver [73,79,80], visceral fat [81], and epicardial fat [82] following treatment with an SGLT2 inhibitor.

In conclusion, administration of SGLT2 inhibitors results in a modest but significant increase in β-cell function and β-cell glucose sensitivity. Long-term studies indicated sustained glucose lowering after at least 2 years of treatment. To the best of our knowledge, no wash-out studies have been conducted to investigate whether improved β-cell function is sustained after stopping treatment. With regard to insulin sensitivity, several research groups reported improved insulin sensitivity, but the improvements were small. It is suggested that the beneficial effects of SGLT2 treatment are mainly caused by a decreased glucotoxicity. However, clinical trials investigating β-cell function and insulin sensitivity over a longer period of time are limited. It could be that treatment for periods longer than 3–4 months may show a different result. For example, data suggest that after 3–4 months, energy losses are compensated by increased food intake that would explain why body weight does not decrease further after this period of time [83,84]. The available data on β-cell function and insulin sensitivity and the fact that SGLT2 inhibitors work independent of insulin suggest that SGLT2 inhibitor therapy could be beneficial in all four proposed novel subgroups of T2D. The first study to investigate the efficacy of SGLT2 inhibition and a GLP-1 receptor agonist in patients with SIDD and SIRD has started to recruit (ClinicalTrails.gov Identifier: NCT04451837).

### 5.2 Glucagon-like peptide-1 (GLP-1) receptor agonists

GLP-1 is a hormone produced by L-cells in the intestine in response to food ingestion, especially to meals with a high content of fat and carbohydrates. GLP-1 administration improves glucose levels through different mechanisms including glucose-dependent insulin secretion, reduction in food intake, reduced body weight, and decreased levels of glucagon. Glucagon-like peptide-1 receptor agonists (GLP-1RA) reduce HbA1c in a range from 0.5 to 1.5% [85,86].

#### 5.2.1 GLP-1RA and β-cell function

One of the expected working mechanisms of GLP-1RA is via a direct effect on β-cells. β-cells express GLP-1 receptors. GLP-1 receptors are G protein-coupled and after activation result in increased cAMP and PKA activity, promoting insulin release from β-cells [87]. The LIBRA trial assessed β-cell function in patients with recent T2D diagnosis treated with insulin for 4 weeks before randomisation with either a long-acting GLP-1RA or placebo for 48 weeks and found improved β-cell function measured by insulin secretion sensitivity index 2 in the active group [88]. Another randomised controlled trial in patients with T2D compared the effect of a short-acting GLP-1RA vs placebo for three years and observed improvements in β-cell function measured by the Mari model, a method that assesses β cell function from values obtained during an OGTT [89].

Anholm et al. found that 12 weeks of metformin plus GLP-1RA led to a significant increase in β-cell function as assessed by the disposition index compared to a metformin or placebo group in a randomised, double-blind crossover trial [90]. Another randomised controlled trial investigated the effect of GLP-1RA plus metformin vs metformin plus lifestyle interventions on β-cell function in patients with recent T2D diagnosis and found that liraglutide improved β-cell function expressed as β-cell secretion during an OGTT compared to a control group within a 15-month period [91]. The positive effects of short- and long-acting GLP-1RA on β-cell function have been demonstrated in several randomised clinical trials.

In animal models of diabetes, it has been shown that GLP-1RA treatment improves the function of β-cells mainly through proliferation and differentiation [92]. However, whether GLP-1RA increases functional β-cell mass in humans is so far unknown. The results of wash-out studies [88,89] showed no lasting effect on β-cell function and therefore indicated that there was no effect on functional β-cell mass and the effects on β-cell function seemed to be acute.

#### 5.2.2 GLP-1RA and insulin sensitivity

Gastaldelli et al. [93] investigated the acute effect of a short-acting GLP-1RA on hepatic and adipose tissue insulin sensitivity measured as glucose and glycerol tracer kinetics after a 13C-enriched glucose load. The study was conducted in patients with T2D and subjects with IGT. They found that acute treatment with GLP-1RA improved hepatic and adipose tissue insulin sensitivity compared to placebo. The prolonged effects of GLP-1RA on insulin sensitivity were assessed by Zander et al. [94]. They investigated the effect of continuous subcutaneous infusion of GLP-1RA vs saline infusion using a portable pump for 6 weeks in patients with T2D and found that insulin sensitivity measured by hyperinsulinaemic euglycaemic clamps increased by 77%. However, this effect on insulin sensitivity could have been overestimated as the study was neither randomised nor blinded. The improvement in insulin sensitivity was accompanied by a decrease in fasting plasma glucose and FFA levels that could have contributed to the effect. Anholm et al. [95] investigated the effect of GLP1-RA plus metformin vs metformin plus placebo on insulin sensitivity in obese and overweight patients with newly diagnosed T2D and coronary artery disease. Insulin sensitivity was measured by the ISI composite, a measurement of whole-body insulin sensitivity obtained from a formula that combines values derived from an OGTT and values from fasting plasma glucose and insulin [96]. GLP1-RA plus metformin increased β-cell function as measured by the disposition index by 40% compared to metformin plus placebo, but insulin sensitivity was not significantly different between the groups [95].

Armstrong et al. [97] evaluated the effect of GLP-1RA on hepatic insulin sensitivity measured as suppression of EGP after 12 weeks of GLP-1RA treatment vs placebo in subjects with non-alcoholic steatohepatitis (NASH). A hyperinsulinaemic euglycaemic clamp was used before and after treatment, and it was found that GLP-1RA reduced EGP compared to placebo (−9.3 vs −2.5%). GLP-1RA also significantly reduced body weight in the intervention group compared to placebo. Dutour et al. [98] evaluated the effect of GLP-1RA on hepatic fat content measured by magnetic resonance spectroscopy (MRS) in obese patients with T2D. After 26 weeks of treatment, they found a significant reduction in hepatic fat content in the intervention group vs placebo (−23.8% vs +12.5%). This reduction in liver fat was highly correlated to body weight loss.

Indeed, the effect of GLP-1RA on body weight may explain the beneficial effects on hepatic and peripheral insulin sensitivity that have been observed. A meta-analysis that included 25 trials comparing GLP-1RA against placebo, insulin, or other glucose-lowering medications found that GLP-1RA led to a significant reduction in body weight [99]. The results showed a mean difference of −2.9 kg body weight loss in an intervention group compared to a control group. Davies et al. [100] also reported the long-term effect on body weight after 56 weeks of treatment compared to placebo in overweight and obese subjects with T2D and reported significantly larger weight loss in an intervention group compared to placebo. Other potential explanations of the effect on insulin sensitivity could be the association that has been found in animal models between GLP-1RA treatment and decreased inflammation [101]. Lynch et al. [102] investigated the association between GLP-1RA therapy and invariant natural killer T (iNKT) cells in human and mice adipose tissue and observed that GLP-1RA activated iNKT cells. Interestingly, iNKT cell activation can lead to weight loss. Therefore, GLP-1RA may partly reduce body weight and improve insulin sensitivity by acting on the immune system.

In conclusion, GLP-1RA improves β-cell function during treatment, but the effect does not persist after discontinuation of treatment [103]. The GLP1-RA treatment effect on glucose control seems to be mainly based on the ability to increase insulin secretion, with contribution of improved insulin sensitivity via weight loss and immune modulatory effects. However, there is limited information on changes in insulin sensitivity after GLP-1RA administration.

Current guidelines establish GLP-1RA as a second-line therapy in obese patients and patients with a diagnosis of CV disease. We suggest that GLP-1RA therapy could also be a preferred treatment option for the obese subgroups described by Ahlqvist, including SIRD, MOD, and SIDD. Considering the initial nausea, GLP-1RA may be a less attractive treatment for the MARD group, considering the age of onset and lower risk of developing diabetes-associated end-organ damage.

### 5.3 Dipeptidyl peptidase 4 (DPP-4) inhibitors

DPP-4 inhibitors are a class of glucose-lowering medications that inhibit the enzyme DPP-4. This enzyme is expressed on the surface of many cells such as adipocytes, kidneys, liver, and small intestine and it decreases the activity of peptides, such as GLP-1 and glucose-dependent insulinotropic polypeptide (GIP). DPP-4 inhibitors properties are characterised by competitive inhibition and high affinity to DPP-4. DPP-4 inhibitors reduce HbA1c in a range of 0.5–1% [104].

#### 5.3.1 DPP-4 inhibitors and β-cell function

DPP-4 inhibitors’ effect on glucose metabolism is thought to be mainly by increasing the availability of incretins such as GLP-1 and GIP, which are responsible for increasing insulin secretion and decreasing glucagon secretion after a meal [105]. The effect on β-cell function was established in several clinical studies. A meta-analysis of 23 randomised placebo-controlled studies associated DPP-4 inhibitor treatment with a significant improvement in HOMA-B compared to placebo [106]. When DPP-4 inhibitors were used as add-on therapy, a significant improvement in HOMA-B was found. HOMA-B is mainly a measure of insulin secretion, and only a few studies have measured the effect of DPP-4 inhibitors on β-cell function using gold standard methods.

In animal models of obesity, treatment with DPP-4 inhibitors for 11 months was associated with better β-cell function measured as the oral disposition index obtained during an OGTT, but not associated with an increase in β-cell mass compared to controls [107]. In humans, Derosa et al. [108] investigated the effect of a DPP-4 inhibitor plus metformin compared to metformin plus placebo on the secretory capacity of β-cells using euglycaemic hyperinsulinaemic and hyperglycaemic clamps combined with subsequent arginine stimulation. They found improved β-cell function expressed as the disposition index after 12 months of DPP-4 treatment (from 163.8 ± 37.9 to 279.5 ± 56.9 nmol/L × μmol/kg) compared to controls (from 163.6 ± 37.7 to 214.2 ± 48.4 nmol/L × μmol/kg).

Although the effects of DPP-4 inhibitors are mainly thought to be via increased incretin levels, Aulinger et al. [109] studied the effects of DPP-4 inhibitors on glucose homeostasis in patients with T2D after blocking GLP-1 action through a GLP-1 receptor antagonist. Interestingly, they found significant effects of DPP-4 inhibitors on insulin secretion during an OGTT despite GLP-1 receptor blockade. Increased GIP action is a possible candidate to explain this independent effect. Yanagimachi et al. [110] measured incretin levels after DPP-4 inhibitor administration during an OGTT in non-diabetic subjects and found that DPP-4 administration not only increased GLP-1, but also bioactive GIP levels.

#### 5.3.2 DPP-4 inhibitors and insulin sensitivity

DPP-4 inhibitors’ effects on insulin sensitivity have been investigated in animal models. For instance, in rats, Pospisilik et al. [111] found an increase in insulin-mediated glucose uptake in muscle tissue and an increase in insulin sensitivity measured by the Matsuda index after treatment with DPP-4 inhibitors compared to controls. Nevertheless, in humans, the effects of DPP-4 inhibitors on insulin sensitivity remain controversial. Derosa et al. [112] evaluated the effects of a DPP-4 inhibitor as an add-on therapy in insulin sensitivity in subjects with T2D and found that HOMA-IR significantly decreased after 12, 18, and 24 months of treatment in an intervention group compared to a control group. However, HOMA-IR does not accurately measure insulin sensitivity in intervention studies. Parthan et al. [113] found no effect of 6 months of DPP-4 inhibitor treatment compared to placebo on insulin sensitivity as measured by hyperinsulinaemic-euglycaemic clamps in well-controlled T2D subjects. These results suggest that, despite a reduction in HbA1c and fasting glucose levels, there seems to be a lack of effects of DPP-4 inhibitors on insulin sensitivity, which contrasts with the effects of GLP-1RA interventions. A possible explanation may be the fact that DPP-4 inhibitors in several studies did not seem to have any significant effects on weight loss [106,114].

Interestingly, in animal models of obesity, weight gain has been associated with an increase in DPP-4 expression in hepatic tissue [115]. In humans, DPP-4 activity has also been associated with a higher BMI, increased fat percentage, and NAFLD [116]. These findings may suggest that DPP-4 inhibition would be a target to reduce hepatic fat content. Indeed, DPP-4 inhibitor treatment in animal models has been associated with improvements in liver steatosis [117,118] and fibrosis [119]. However, in humans, treatment with DPP-4 inhibitors has not shown any effect on NAFLD [120,121].

In conclusion, DPP-4 inhibitors have a significant effect on insulin secretion compared to placebo and probably their main effect on glucose control is via increasing insulin secretion rather than having an effect on insulin sensitivity. DPP-4 inhibitors compared to GLP-1RA treatment seem to have no effect on body weight and may therefore be less favourable for patients who mainly benefit from weight loss. We suggest that DPP-4 inhibitor therapy could be a treatment option for SIDD and MARD because of the lack of DPP4 inhibition on body weight and insulin resistance.

### 5.4 Thiazolidinedione

Thiazolidinediones, also known as glitazones, belong to the group of insulin sensitizers. Thiazolidinediones were first discovered by screening for a hypoglycaemic effect in ob/ob mice [122]. It was later discovered that thiazolidinediones improved insulin sensitivity in insulin-resistant animal models. In humans, similar results were found, as thiazolidinedione administration caused a reduction in glucose and insulin levels and improved insulin resistance and lipid metabolism. It is generally accepted that thiazolidinediones act as a nuclear peroxisome proliferator-activated receptor (PPAR) agonist specifically for the gamma subtype (PPAR-γ) that is predominantly expressed in white adipose tissue but to a lesser extend in the muscle, liver, and heart [123,124]. Activation of PPAR-γ results in transcription of the PPARγ target genes that are mainly involved in lipid and carbohydrate metabolism and immune functions [[125], [126], [127]]. Due to severe adverse effects, most types of thiazolidinediones, including troglitazone and rosiglitazone, have been withdrawn from the market. Only pioglitazone is currently approved by the European Medicines Agency (EMA) and the US Food and Drug Administration (FDA) to treat T2D, and we therefore focus solely on thiazolidinedione in this review. In general, pioglitazone administration is associated with plasma glucose reductions of 1.2–2.0 mmol/L, HbA1c reductions of 0.9–1.3%, and an increase in body weight of 3.6 kg [128,129].

#### 5.4.1 Thiazolidinediones and β-cell function

The effect of pioglitazone on β-cell function was established in a meta-analysis [130]. With monotherapy, HOMA-B improved by 16% compared to baseline. When pioglitazone was combined with metformin or sitagliptin (a DPP-4 inhibitor), a small but significant improvement of 9.8 and 11.8% in HOMA-B, respectively, was observed in patients with T2D. However, although HOMA-B provides some information about the effect of pioglitazone on β-cell function, trials using the gold standard to assess β-cell function, the disposition index, are limited. To the best of our knowledge, only 2 clinical trials reported β-cell function assessed via the disposition index in patients with T2D. Gastaldelli et al. [131] and Tripathy et al. [132] reported improved β-cell function measured by the disposition index after pioglitazone administration for 4 and 6 months, respectively. How pioglitazone improves β-cell function is unknown, but it may involve direct (expression of PPAR-γ in pancreatic islet cells [133]) or indirect effects related to marked improvements in insulin sensitivity by pioglitazone.

Over a longer period of time as measured in the PROactive trial with an average follow up of 34.5 months, pioglitazone was more effective at reducing HbA1c levels than placebo in patients treated with either metformin or sulfonylureas. The reduction in HbA1c occurred rapidly and was sustained over the full period of time [134], indicating the long-term effect of pioglitazone on preserving β-cell function.

#### 5.4.2 Thiazolidinediones and insulin sensitivity

The effects of thiazolidinediones on insulin sensitivity in humans have been extensively studied and reviewed. In a systematic review, Natali and Ferrannini [135] identified 23 papers that measured the effects of thiazolidinediones on peripheral glucose disposal by hyperinsulinaemic clamps and/or EGP using glucose tracer analyses in patients with T2D. A combined data analysis revealed improvements in a range of 31–36% and 19–33% in peripheral and hepatic insulin sensitivity, respectively, after thiazolidinedione administration compared to baseline or placebo. However, in this systematic review, not only pioglitazone was included, but also troglitazone and rosiglitazone.

With respect to pioglitazone, several research groups showed a statistically significant improved peripheral [131,[136], [137], [138], [139], [140], [141], [142], [143], [144], [145], [146]], hepatic [131,[143], [144], [145], [146]], and adipose tissue [137,145,147] insulin sensitivity in patients with T2D.

Because PPARγ is predominantly expressed in adipose tissue, it is suggested that improvements in peripheral and hepatic insulin sensitivity as well as β-cell function are indirect and mainly elicited by a decreased flux of fatty acids from adipose tissue, increasing insulin-mediated glucose uptake and reducing lipotoxicity. It is well known that PPARγ activation by pioglitazone leads to reduced plasma levels of triglycerides and FFA [148,149]. Since higher FFA levels are associated with ectopic fat accumulation and insulin resistance, decreasing FFA probably plays an important role in improving insulin sensitivity. Indeed, pioglitazone administration is associated with a redistribution of adipose tissue resulting in reduced ectopic and visceral lipid storage, but increasing subcutaneous adipose tissue. Promrat et al. [150] were the first group to describe the effects of pioglitazone administration on hepatic lipid content in non-diabetic patients with NASH. In this trial, hepatic lipid content decreased significantly from 47.5% to 22.8% after 48 weeks of pioglitazone administration, but the total body fat percentage increased from 35.8% to 37.6%. The insulin sensitivity index assessed during a frequently sampled intravenous glucose tolerance test improved. Rasouli et al. [140] investigated the effects of pioglitazone vs metformin administration for 10 weeks on insulin sensitivity and intramyocellular lipid content (IMCL) in patients with IGT. They reported a significant decrease in IMCL after pioglitazone compared to metformin therapy and baseline. This lowering of IMCL content was accompanied by an increase in insulin sensitivity assessed via an insulin-modified intravenous glucose tolerance test, with a redistribution of visceral fat toward subcutaneous fat stores.

Similar results were later reported by several research groups. Pioglitazone administration in patients with prediabetes and T2D co-treated with dietary advice, hypocaloric diets, metformin, or insulin led to a decrease in hepatic [145,[151], [152], [153], [154]], intramyocellular [152], and myocardial [154] lipid content and an increase in subcutaneous fat [145,152,154]. Despite reduced ectopic fat, treatment causes an increase in body weight. This increase in body weight is the result of a higher caloric intake in patients treated with pioglitazone [155].

Of note, not all studies were consistent with pioglitazone's effect on metabolic adaptations. Phielix et al. [147] reported improved adipose tissue insulin sensitivity but did not find improved peripheral or hepatic insulin sensitivity despite a decrease in hepatic lipid content after 12 weeks of pioglitazone therapy in non-obese patients with T2D. Van der Meer et al. [156] reported decreased hepatic lipid content but no changes in intramyocardial lipid content or myocardial fatty acid oxidation after 24 weeks of pioglitazone administration in patients with T2D. Bajpayi et al. [136] reported a significant shift from IMCL toward extramyocellular lipid (EMCL) in the gastrocnemius, tibialis anterior, and soleus muscles and a tendency toward a decrease in hepatic lipid content after 12 weeks of pioglitazone administration in patients with T2D. These changes were accompanied by an improved peripheral insulin sensitivity and metabolic flexibility (Δrespiratory quotient) measured during insulin infusion (80 mU/min/m^2^) compared to the fasted state of a hyperinsulinaemic-euglycaemic clamp. Substrate oxidation in the fasted state and mitochondrial function assessed as resting ATP turnover and the maximal ATP synthetic rate by 31P-MRS were unaffected by pioglitazone.

In conclusion, pioglitazone is effective at reducing peripheral, hepatic, and adipocyte insulin resistance mainly via the amelioration of lipotoxicity by reducing ectopic lipid storage. Pioglitazone is also effective at lowering HbA1c over a longer period, reflecting improved β-cell function. However, these effects are not sustained after discontinuation of pioglitazone [157]. Pioglitazone can be a powerful treatment in a limited group of patients, where improvements in insulin resistance and NAFLD outweigh side effects such as weight gain, osteoporosis [158], and water retention, increasing the risk of heart failure [159]. We hypothesise that pioglitazone could be a beneficial treatment for SIDD and SIRD and should be avoided in patients with MOD and MARD due to the side effects.

## 6 Concluding remarks

T2D is a heterogeneous disease with a complex metabolic disarray leading to hyperglycaemia and progressive β-cell dysfunction. Several second-line treatment options currently exist; however, finding the most optimal type of medication for patients with T2D can be challenging. The classification system suggested by Ahlqvist et al. provides a spectrum of the disease that enables more insight into the underlying metabolic causes of T2D.

Based on the reported effects of the currently available anti-diabetic medications on β-cell function, insulin sensitivity, and metabolism, some medications may be more suitable for treating subgroups of patients. Metformin is the recommended first-line therapy for glucose control of patients with T2D and possibly works as a first-line therapy for patients in all four T2D subgroups. Metformin may even be sufficient as the only therapy in patients with mild disease, mainly including some with MARD and MOD. However, for patients with severe insulin deficiency (SIDD), we conclude that they could benefit from most of the current second-line anti-diabetic treatments. Since the SIDD group is associated with a lower BMI, there is also no preferred type of medication for those patients to correct body weight.

Patients with severe insulin resistance (SIRD) characterised by a higher BMI and prevalence of NAFLD may benefit most from treatments that reduce body weight and improve insulin sensitivity. This group of medications includes SGLT2 inhibitors because of the potential improvements in insulin sensitivity and clinically relevant reductions in body weight. Whether GLP-1RA treatment is beneficial in this group remains unclear due to the limited clinical trials investigating insulin sensitivity. However, as GLP-1RA reduces body weight and hepatic lipid content, this treatment option could be beneficial for SIRD. Pioglitazone treatment is also effective for improving insulin sensitivity and reducing NAFLD, but should only be considered when no other treatment options are available because of the well-established weight gain and other adverse effects associated with pioglitazone administration. DPP-4 inhibitors do not seem to play a therapeutic role in this group as there are no established effects on insulin sensitivity, weight reduction, or NAFLD.

Patients with mild obesity-related diabetes (MOD) are characterised by moderate insulin resistance and mild insulin deficiency, but a high BMI. This particular group may benefit from treatment with metformin only, but if it cannot control glucose levels, they could benefit the most from GLP-1RA and SGLT2 inhibitor treatment, as both medications significantly reduce body weight. Treatment with pioglitazone should be avoided in this group because of the weight gain associated with this medication.

For patients with mild age-related diabetes (MARD) who are characterised by moderate insulin resistance and mild insulin deficiency, older age at the time of diagnosis, and lower risk of developing end-organ damage, sulfonylurea and DPP-4 inhibitors could be the best options as additional therapies if metformin treatment alone cannot control glucose. However, SGLT2 inhibitors and GLP-1RAs may also be an option in MARD patients with established end-organ diseases such as CV disease and reduced kidney function. Since this population is older, add-on treatment decisions should be made carefully and possible side effects of each type of medication should always be considered.

To achieve adequate T2D therapy, in this study, we considered that an important proportion of patients will require additional medication on top of lifestyle recommendations and metformin treatment. The subgroups proposed by Ahlqvist et al. and the known metabolic effects on β-cell function and insulin sensitivity of the different classes of medication may provide a more personalised treatment for patients with T2D based on the main underlying causes of hyperglycaemia in each individual as previously outlined. However, the final treatment choice in T2D patients should also consider other factors associated with diabetes. For example, in the presence of CV disease, GLP-1RA or SGLT2 inhibitors are the preferred options without considering their associated subgroups. Other factors such as the presence of kidney disease, the importance of weight loss in combination with lifestyle interventions, patient age, preferences, and potential side effects should be also weighted.

We are aware that the Ahlqvist clustering of subgroups taking into account the metabolic phenotype of T2D may not be the final diabetes classification and more investigations are needed. Furthermore, there are currently no intervention trials providing scientific evidence indicating which anti-diabetic medication is best for patients depending on their metabolic phenotype. Therefore, the suggestions described in this review are hypothesise generating and should not be regarded as recommendations. To establish the most appropriate therapy, future intervention trials are needed in diabetes subgroups to provide a scientific basis for developing personalised medicine to treat the large and diverse populations of patients with T2D.

## Author contributions

All of the authors contributed to writing the manuscript and approved the final version.
